# Test-and-treat coverage and HIV virulence evolution among men who have sex with men

**DOI:** 10.1093/ve/veab011

**Published:** 2021-02-10

**Authors:** Sarah E Stansfield, Joshua T Herbeck, Geoffrey S Gottlieb, Neil F Abernethy, James T Murphy, John E Mittler, Steven M Goodreau

**Affiliations:** Department of Biomedical Informatics and Medical Education, University of Washington, Seattle, WA 98195, USA; Department of Global Health, University of Washington, Seattle, WA 98195, USA; Departments of Medicine & Global Health, University of Washington, Seattle, WA 98195, USA; Department of Biomedical Informatics and Medical Education, University of Washington, Seattle, WA 98195, USA; Department of Microbiology, University of Washington, Seattle, WA 98195, USA; Department of Microbiology, University of Washington, Seattle, WA 98195, USA; Department of Anthropology, University of Washington, Seattle, WA 98195, USA

**Keywords:** HIV, MSM, SPVL, evolution, test-and-treat, mathematical modeling

## Abstract

HIV set point viral load (SPVL), the viral load established shortly after initial infection, is a proxy for HIV virulence: higher SPVLs lead to higher risk of transmission and faster disease progression. Three models of test-and-treat scenarios, mainly in heterosexual populations, found that increasing treatment coverage selected for more virulent viruses. We modeled virulence evolution in a population of men who have sex with men (MSM) with increasing test-and-treat coverage. We extended a stochastic, dynamic network model (EvoNetHIV). We varied relationship patterns (MSM vs. heterosexual), HIV transmission models (increasing vs. plateauing probability of transmission at very high viral loads), and treatment roll-out (with explicit testing or fixed intervals between infection and treatment). In scenarios most similar to previous models (longer relational durations and the plateauing transmission function), we replicated trends previously found: increasing treatment coverage led to increased virulence (0.12 log_10_ increase in mean population SPVL between 20% and 100% treatment coverage). In scenarios reflecting MSM behavioral data using the increasing transmission function, increasing treatment coverage selected for viruses with *lower* virulence (0.16 log_10_ decrease in mean population SPVL between 20% and 100% treatment coverage). These findings emphasize the impact of sexual network conditions and transmission function details on predicted epidemiological and evolutionary outcomes. Varying these features creates very different evolutionary environments, which in turn lead to opposite effects in mean population SPVL evolution. Our results suggest that, under some realistic conditions, effective test-and-treat strategies may *not* face the previously reported tradeoff in which increasing coverage leads to evolution of greater virulence. This suggests instead that a virtuous cycle of increasing treatment coverage and diminishing virulence is possible.

## 1. Introduction

Pathogen evolution in the face of control measures presents major public health challenges. HIV, like other retroviruses, can evolve extremely rapidly in both virulence (harm to host) and transmission risk. HIV evolution following global treatment scale-up could increase the frequency of more virulent forms, subsequently requiring more intense precautions and control measures. While this issue has been considered in heterosexual populations (Roberts, Goulder, and McLean 2015; Herbeck et al. 2016), to our knowledge no published models explore it specifically among populations of men who have sex with men (MSM), who account for ∼70 percent of new infections in the United States (Centers for Disease Control and Prevention, 2019a).

Relationship dynamics impact the evolutionary landscape for HIV within and across populations. MSM report higher prevalence of temporally overlapping (concurrent) partnerships and higher lifetime numbers of partners than do heterosexuals (Glick et al. 2012). Short mean relationship durations or high proportions of individuals with multiple concurrent partnerships allow individuals to become infected from one partner and transmit to another before leaving the highly infectious early stage of infection (Powers et al. 2011; Goodreau et al. 2012; Leung, Powers, and Kretzschmar 2017; Goodreau et al. 2018b). When more transmissions occur early in the course of infection due to relationship patterns, highly virulent viruses gain an evolutionary advantage, so that modeled populations with either of these traits tend to have higher mean population set point viral loads (MPSPVLs; Goodreau et al. 2018b). This suggests that we should not automatically extrapolate findings about HIV virulence evolution from heterosexuals to MSM.

HIV virulence can be tracked with the proxy measure of set point viral load (SPVL), the viral load (VL) established shortly after initial infection. SPVL influences both disease progression and transmission potential: higher VLs are more likely to transmit (Fraser et al. 2007; Hughes et al. 2012) and higher SPVLs are associated with faster progression to AIDS-defining illnesses (Modjarrad, Chamot, and Vermund 2008). Because HIV SPVL is partially heritable from the infecting partner (Alizon et al. 2010), variable among individuals (Fraser et al. 2007), and associated with differences in transmission rate (viral fitness), it fulfills the conditions for evolution via natural selection, and can be viewed through the framework of evolutionary tradeoffs (Ewald 1983, 2011).

The “virulence-transmission trade-off” hypothesis suggests that a pathogen’s transmission rate is linked to the duration of infection (Anderson and May 1982; Ewald 1983). HIV follows the trade-off curve constraint, as time to AIDS-defining illness and probability of transmission both depend on SPVL (Fraser et al. 2007; Modjarrad, Chamot, and Vermund 2008; Hughes et al. 2012). Other theories on the evolution of virulence consider pathogen transmission mechanism, as pathogens that require direct contact between hosts and susceptible individuals should allow the host to remain mobile longer than other pathogens (Ewald 1983, 2011). Sexually transmitted infections including HIV follow this pattern, as they are generally benign in the early stages of infection but may be fatal in later years.

The evolution of HIV virulence occurs in the context of changing VL and transmission probability across multiple stages of infection. In the acute phase (∼3 months) and early infection phase, VL and transmission probability rapidly increase as the virus replicates, then decrease to an individual-specific SPVL (Fraser et al. 2007; Hughes et al. 2012). The chronic phase begins after SPVL is achieved. VL increases gradually over this phase, which has an estimated median duration of 9.4 years (95% CI 8.7, 10.0) in MSM (Babiker et al. 2000) in the absence of antiretroviral treatment (ART). Higher SPVLs are associated with both shorter time in this phase and higher risk of transmission (Modjarrad, Chamot, and Vermund 2008). Finally, in the AIDS phase VL increases as the immune system can no longer control virus replication.

The opportunities for transmission across these stages can influence how virulence evolves (Goodreau et al. 2018b). For example, it may be more advantageous for a virus with numerous transmission opportunities in the acute and early chronic phases to evolve high virulence and a greater chance of transmitting quickly than to have lower virulence and allow the host to live longer (Goodreau et al. 2018b). Changes in the infections occurring in the AIDS phase have less impact on SPVL evolution while still influencing prevalence and incidence (Goodreau et al. 2018b).

With treatment, an individual’s VL can become undetectable, with little or no risk of onward transmission (Rodger et al. 2016) or progression to AIDS (Sterne et al. 2005). Since 2015, treatment guidelines have recommended immediate ART initiation following positive diagnosis, also known as test-and-treat (World Health Organization, 2015; DHHS Panel on Antiretroviral Guidelines for Adults and Adolescents 2016). The proportion of persons living with HIV (PLWH) who are durably virally suppressed in the US is estimated at 61.5 percent and rising (Centers for Disease Control and Prevention, 2019b), increasing the importance of considering the effects of large-scale HIV treatment on virulence evolution.

Numerous studies have modeled the effects of large-scale test-and-treat campaigns on epidemiological outcomes such as incidence (Granich et al. 2009; Hontelez et al. 2013; Kretzschmar et al. 2013; Eaton et al. 2015), but few articles explore these effects on virulence evolution (Roberts, Goulder, and McLean 2015; Herbeck et al. 2016; Smith and Mideo 2017). The heterosexual models looked at the effects of ART rollout in sub-Saharan Africa. Roberts, Goulder, and McLean (2015) examined virulence change with widespread test-and-treat campaigns with a simple deterministic two-strain model. They found that the more virulent strain predominated in all scenarios that did not lead to virus extinction. Herbeck et al. (2016) developed a stochastic agent-based test-and-treat model that examined behavioral parameters with a primary model based on a setting with a core group of individuals with a higher act rate, and an alternate model with random mixing. Mean SPVL increased with higher levels of treatment in both models. Smith and Mideo (2017) used a compartmental model with simplified HIV dynamics and the added complexity of “leaky” therapy, in which agents receiving treatment can still transmit infection. The increasing use of fully suppressive treatment caused MPSPVL to increase and, when treatment was leaky, for SPVL to evolve even higher. Thus, the existing literature consistently predicts increasing virulence with increased treatment, across a range of assumptions. As mentioned, however, none of these models explicitly consider MSM populations, which have different relational dynamics, interacting with higher per-act transmission probabilities, as well as relatively high mean levels of testing (Hall et al. 2017) and ART use (Centers for Disease Control and Prevention, 2019a).

**Figure 1.**
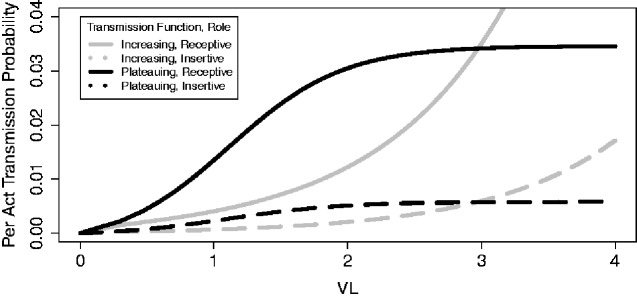
The plateauing (Fraser et al. 2007) (black) and exponentially increasing (Hughes et al. 2012) (gray) transmission functions, as implemented for MSM in EvoNetHIV. The exponentially increasing function was converted from Hughes et al.’s (2012) original function (full details in the Supplementary Appendix). The role in the legend reflects that of the negative partner.

Additionally, when modeling HIV spread one must select a mathematical function that relates VL to the probability of transmission. Fraser et al. (2007) and Hughes et al. (2012) both estimated transmission functions based on studies of serodiscordant heterosexual couples. The resulting two estimated functions have different forms, as the former plateaus at high VLs (Fig. 1, black lines) while the latter increases exponentially over the range of VLs typically found in PLWH (Fig. 1, gray lines). We refer to these as the plateauing and increasing functions, respectively. Some of the difference in shape in transmission functions arises through the paucity of data about transmission at high VLs, along with small samples sizes (86 and 129 linked transmissions in the cohorts on which the increasing and plateauing functions were based, respectively (Fideli et al. 2001; Lingappa et al. 2010)), methodological differences, and noisy data (see Supplementary Appendix for further discussion). Differences in the shape of these functions can have large impacts on the likelihood of transmission at different stages of infection. The extent to which these possible relationships between VL and transmission might influence the evolution of virulence in the face of test-and-treat campaigns is an important question; thus far, only the plateauing function has been used in these models (Herbeck et al. 2016; Smith and Mideo 2017). For example, very high virulence becomes relatively less advantageous with the plateauing function, as in that function the higher SPVLs only result in shorter times to AIDS but little corresponding increase in transmission probability.

In this work, we model the patterns of virulence evolution that occur with increasing proportions of test-and-treat coverage with parameters based on the previous literature (Herbeck et al. 2016), qualitatively confirming whether we can replicate previous predictions about virulence evolution using similar assumptions. In order to better understand our modeling assumptions, we then examined a series of models that varied each assumption alone and in combination, including factors that may vary between populations (treatment initiation timing and behavioral assumptions) and intrinsic biological factors (the transmission scenario: plateauing vs. increasing). We consider the implications for the expansion of test-and-treat among MSM populations and for future research.

## 2. Methods

We extended EvoNetHIV, a stochastic, dynamic, network-based model previously described (Herbeck et al. 2018; Goodreau et al. 2018b; Stansfield et al. 2019), to predict MPSPVL, incidence, and prevalence changes with increasing levels of test-and-treat coverage in an MSM population. This package builds on the EpiModel (Jenness, Goodreau, and Morris 2018) and *statnet* (Handcock et al. 2008) R packages and is available at github/EvoNetHIV/Test_and_Treat. Full methods for this model are detailed in Supplementary Appendix.

Parameters for sexual network structure, agent attributes, and behavior (excluding relationship duration and coital frequency) were acquired from studies of Atlanta MSM (Hernández-Romieu et al. 2015; Sullivan et al. 2015) as derived in Goodreau et al. (2018a). Men were exclusively insertive (24% of agents), exclusively receptive (27%), or role versatile (49%, Goodreau et al. (2018a)). Men with incompatible sexual roles (i.e. two exclusively receptive men) could not form a partnership. SPVL was fixed for each individual, while current VL varied with time and treatment status. CD4+ cell count and rate of progression to AIDS depended on SPVL and treatment status. Separable temporal exponential random graph models, as implemented in *statnet*, were used to model sexual network structure (Krivitsky and Handcock 2014). Mean momentary degree, the average number of relationships a man is in at a cross-section of time, was 0.70 (Goodreau et al. 2018a). Within partnerships, coital acts occurred stochastically and ceased during the latter half of the AIDS phase. Condom use per act occurred stochastically with 50 percent probability (Goodreau et al. 2018a). Relationships dissolved with a constant hazard based on relationship duration.

Given a serodiscordant act, transmission depended on donor’s current VL, each partner’s sexual role, condom use, and the HIV-negative partner’s circumcision status (if he was the insertive partner). Individual SPVL has heritability of 0.36 (Hollingsworth et al. 2010); that is, the model calculates an individual’s SPVL using two additive actors such that 36 percent of the variation in SPVL in our model can be explained by variation in donor SPVL. The heritable factor came from the infecting partner with the addition of a random mutational parameter, while the nonheritable factor followed a normal distribution representing environmental and host contributions. Initial population MPSPVL was 4.5 log_10_ copies/ml (Herbeck et al. 2012).

Treatment began ten years after modeled time began to allow for model burn-in, then continued to the end of modeled time. Treatment coverage ranged from 0 to 100 percent of the eligible population in 20 percent increments. We modeled incomplete coverage by assigning an individual attribute (“treatable”) with probability dependent on the scenario’s coverage. With treatment, an individual’s VL exponentially decayed and became undetectable. We did not model incomplete adherence or incomplete viral suppression.

The initial population included 10,000 individuals 18–55 years old. HIV prevalence at the beginning of the simulation was 10 percent. Simulations lasted forty years after treatment began with one-day time steps. Arrival into the model population followed a Poisson distribution giving 1 percent annual population growth. Departures ensued through aging out of the population, AIDS mortality, and background mortality. We tracked viral virulence evolution with MPSPVL change through time and epidemiological factors of prevalence and incidence.

We began by creating a network model designed to share many parameters with the alternate model in Herbeck et al. (2016), while incorporating MSM probabilities of HIV transmission. We selected this model as it corresponded closely to many of the models that previously examined test-and-treat’s impacts on the HIV epidemic. Our initial analysis (Model 1 A-fixed treatment interval, Table 1) included treatment that began at a fixed interval after infection, long mean relationship durations, high mean coital frequencies, and the assumption of transmission probabilities that plateau at higher VLs, all parameterized from Herbeck et al. (2016). We compared these results to prior models to determine whether we replicate the direction of effect.

**Table 1.** Model parameter table. The main analysis consisted of the models with explicit testing; 64 replicates were completed for each parameter set here.

Model	Transmission function	Relationship duration (months)	Coital frequency (acts/day)	Treatment scheme	Treatment coverage	Test interval, main analysis (years)	Test interval, sensitivity analyses^a^ (years)
Model 1A fixed treatment interval^a^	Plateauing	30.0	1.0	Fixed interval	0–100%, 20% increments	2	1–6, 1 year increments
Model 1B fixed treatment interval^a^	Plateauing	3.3	0.2	Fixed interval	0–100%, 20% increments	2	1–6, 1 year increments
Model 2A fixed treatment interval^a^	Increasing	30.0	1.0	Fixed interval	0–100%, 20% increments	2	1–6, 1 year increments
Model 2B fixed treatment interval^a^	Increasing	3.3	0.2	Fixed interval	0–100%, 20% increments	2	1–6, 1 year increments
Model 1A explicit testing	Plateauing	30.0	1.0	Explicit testing	0–100%, 20% increments	2	1–6, 1 year increments
Model 1B explicit testing	Plateauing	3.3	0.2	Explicit testing	0–100%, 20% increments	2	1–6, 1 year increments
Model 2A explicit testing	Increasing	30.0	1.0	Explicit testing	0–100%, 20% increments	2	1–6, 1 year increments
Model 2B explicit testing	Increasing	3.3	0.2	Explicit testing	0–100%, 20% increments	2	1–6, 1 year increments

a Analyses varying the test interval and treatment scheme were completed with 16 replicates for each parameter set (results in Supplementary Appendix)..

We proceeded to interrogate the model assumptions to determine each feature’s impact on virulence evolution. We varied each feature (treatment initiation, sexual behavior, and transmission function) alone and in combination. Treatment initiation occurred with two methods. In the fixed treatment interval method, individuals who were HIV+ and within the treatable proportion of the simulation began treatment at intervals ranging from 1 to 6 years after infection, in 1-year increments. With the explicit testing method, if an agent was HIV+, had a detectable VL, and was within the treatable proportion of the simulation, they began treatment immediately after testing positive to simulate an explicit test-and-treat scheme. Mean test intervals ranged from 1 to 6 years, in 1-year increments. Sexual behaviors reflected the previous modeling literature (Models 1A and 2A) or MSM behaviors derived from data (Models 1B and 2B), each described above. In the reported data, mean relationship durations were shorter and coital frequency was lower (Table 1). As mean momentary degree remained constant, there was a greater total number of relationships when mean relationship durations were shorter (mean lifetime partners = 8.96 in Models 1A and 2A and 81.45 in Models 1B and 2B). These numbers are roughly in line with a comparative analysis of lifetime partners for both groups (Glick et al. 2012). In the oldest age group compared (30–39-year-olds, who have of course not all finished acquiring new partners), median lifetime number of new partners was 7 for heterosexual women and 12 for heterosexual men (whose behavior is reflected in Models 1A and 2A for comparison with previous models), and 67 for MSM (reflected in models 1B and 2B). However, there were greater mean lifetime sex acts in Models 1A and 2A (8,064 versus 1,612.71 in Models 1B and 2B), given that the models were are comparing to in the former effectively assumed daily sex, while Models 1B and 2B use rates derived from MSM data. The transmission function relating the VL of the HIV+ partner to the probability of HIV transmission followed the plateauing (Models 1A and 1B) or increasing (Models 2A and 2B) patterns as described above.

We present most variable values for years 20–40, the second half of modeled time after treatment began. This allows sufficient MPSPVL evolution to occur to make effects apparent. We calculated 95% confidence intervals for variables of interest in each parameter set. While we recognize that calculating inferential statistics for simulated data is a topic for debate, we believe it can be valuable if the number of replicated simulations is fixed in advance, especially at levels comparable to the number of observations in a typical empirical study of similar phenomena. This follows Ferguson et al. (2006); Yang et al. (2009); Goodreau et al. (2018b); and others.

## 3. Results

**Figure 2.**
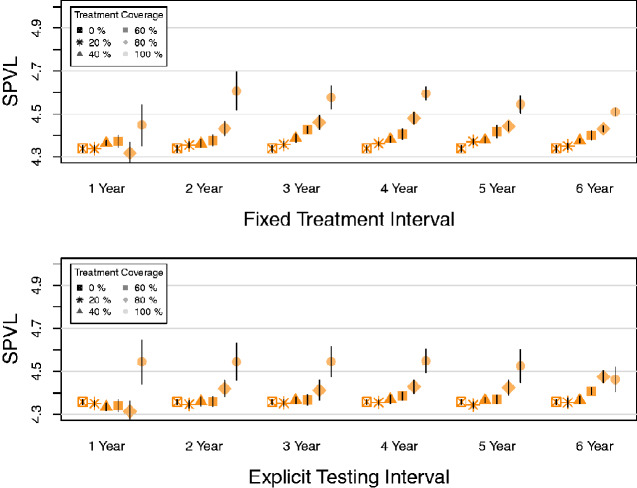
Fixed treatment interval and explicit testing impact on MPSPVL in Model 1A. The fixed interval between infection and treatment and the mean explicit testing interval were varied from 1 to 6 years. Treatment began in year 0. Each symbol is the mean of sixteen simulations. Bars show 95% confidence intervals. (a) Mean population SPVLs of those infected in years 20–40 in simulations using fixed interval treatment initiation. (b) Mean population SPVLs of those infected in years 20–40 in simulations using explicit testing treatment initiation.

The form of treatment initiation (fixed treatment interval or explicit testing) had little qualitative impact on MPSPVL evolution (Fig. 2a vs. b). Given this, we focus on explicit testing results for all analyses as this scenario models clinical practice explicitly; further results featuring fixed interval treatment are detailed in Supplementary Appendix. We focus on two-year testing intervals in the remaining results, as MPSPVL evolution patterns were clearest here while qualitatively similar to those found at other intervals.

As expected, increasing test-and-treat proportions led to decreased incidence and prevalence. In the absence of treatment, incidence and prevalence varied substantially among models. Models 1A and 1B, with the plateauing transmission function, yielded the largest epidemic: at year 40 with no treatment, mean prevalence was 47.9 and 46.4 percent, respectively, and mean yearly percent incidence equaled 9.6 and 9.5. In contrast, Models 2A and 2B, with the increasing transmission function, had mean prevalence equal to 32.5 and 13.1 percent and mean yearly percent incidence equal to 5.5 and 2.2, respectively. Prevalence and incidence in all scenarios decreased with increasing treatment proportions, but the magnitude of this decrease varied between scenarios.

Incident MPSPVL also changed through time depending on the model parameters, showing the evolution of viral virulence. These changes reflected both trends with increasing treatment coverage as well as overall differences between models.

**Figure 3.**
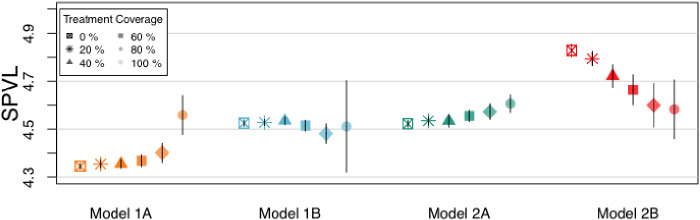
Mean population SPVLs of those infected in years 20–40. Treatment began in year 0. Each symbol is the mean of 64 simulations. Bars show 95% confidence intervals.

Models 1A and 2B produced qualitatively different results with respect to our key question of interest: the direction of SPVL evolution in the face of increasing test-and-treat coverage (Fig. 3). In Model 1A, higher coverage led to higher MPSPVL (MPSPVL = 4.35 log_10_ copies/ml (CI 4.34, 4.37) at 20 percent coverage and 4.56 log_10_ copies/ml (CI 4.48, 4.64) at 100 percent coverage, 0.20 log_10_ copies/ml increase). This result replicated the direction of effect in Herbeck et al. (2016) but differed in magnitude. In contrast, in Model 2B, increasing coverage selected for viruses with lower MPSPVLs (MPSPVL = 4.79 log_10_ copies/ml (CI 4.76, 4.82) at 20 percent coverage and 4.58 log_10_ copies/ml (CI 4.46, 4.71) at 100 percent coverage, 0.21 log_10_  *decrease*). The remaining two models had much less MPSPVL change with increasing treatment (0.02 log_10_ copies/ml decrease and 0.07 log_10_ copies/ml increase between the 20 and 100 percent treatment scenarios in Model 1B and Model 2A respectively). Patterns in MPSPVL change with increasing treatment proportion were not all linear; there was a threshold effect in Model 1A.

Factors that influenced trends in MPSPVLs with increasing treatment coverage included timing of transmission (plots in Supplementary Appendix). Model 1A had the largest increase in the proportion of transmissions occurring in the infecting partner’s acute phase, as 13.8 percent (CI 13.6,14.1) of transmissions occurred there with 20 percent treatment coverage while 26.8 percent (CI 23.1, 30.5) occurred with 100 percent treatment coverage. In contrast, there was little difference in Model 2B with increasing treatment coverage (7.1% (CI 6.8, 7.4) with 20% treatment coverage, 5.4% (CI 2.4, 8.5) with 100% treatment coverage). Models 1B and 2A had small and moderate increases here respectively. The proportion of transmissions that occurred in the infecting partner’s AIDS phase showed mixed effects. Models 1A and 2A exhibited a linear decline with increasing treatment coverage. However, Models 1B and 2B exhibited a threshold effect with little decrease until the 80 or 100 percent treatment coverage levels.

## 4. Discussion

As test-and-treat coverage expands around the world, understanding how HIV virulence will respond to this expansion, especially in highly affected groups such as US MSM, is important in order to predict incidence changes and potential clinical consequences for those not on treatment. The impact of expanding treatment campaigns in reducing new infections could be diminished if increasing test-and-treat coverage caused virulence to increase.

**Figure 4.**
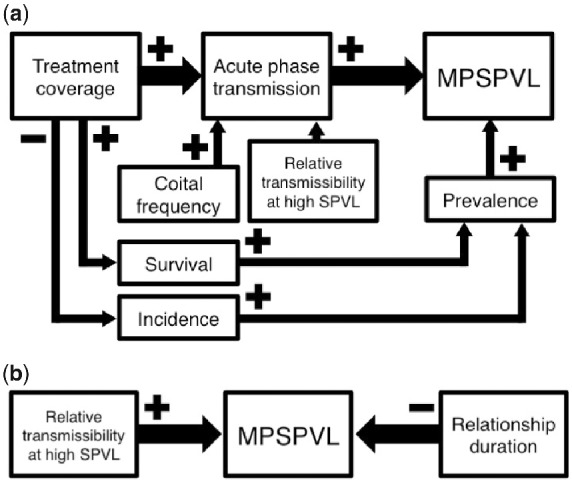
Causal diagram showing the hypothesized relationships between model factors and (a) MPSPVL change and (b) overall MPSPVL. Arrow size shows strength of the effect. The relative transmissibility at high SPVL refers to the probability of transmission at high VLs. In (a) the plateauing transmission function creates more variability in the proportion of transmissions in the acute phase than does the increasing transmission function. In (b) the increasing transmission function has high transmissibility and subsequently higher MPSPVLs while the plateauing transmission function has lower transmissibility and lower MPSPVLs.

In contrast to the previous literature, however, one of our models predicted that HIV virulence would decrease with higher test-and-treat coverage. This model reflected empirical MSM behavioral data and the assumption of an exponentially increasing transmission function. To investigate the results of this and other model assumptions, we varied parameters relating to treatment initiation, sexual behaviors, and transmission function. We found that the impact of these parameters on overall MPSPVL varied depending on the stage in the disease course. To interpret these findings, we first consider Model 1A, then each other model, changing one factor at a time. Fig. 4a lays out the many factors we considered in a causal diagram with symbols indicating the nature of the relationships; we address each factor in turn.

### 4.1 Virulence trends with increasing treatment coverage

In Model 1A, increasing treatment coverage led to increasing virulence (Fig. 3). As higher proportions of individuals were treated, more transmissions shifted to occur early in the course of infection, before treatment initiation. This model had extremely high coital frequency (mean = 1 act/day), so early transmission was more probable given the high number of opportunities. Treatment disproportionately removes people in the later stages of HIV from the infectious population, as they have a higher probability of having tested positive and becoming virally suppressed. As discussed above, shifting transmissions to early infection selects for more virulent viruses. Model 1A had a much larger change in the proportion of transmissions occurring in the acute phase than the other models, so it shows the effect of this shift most clearly.

Model 1B shared the plateauing function with Model 1 A but had a much lower coital frequency. This led to fewer transmission opportunities in the acute phase. While there was a small increase in acute phase transmissions, the effect on MPSPVL was balanced by the large difference in prevalence between the treatment coverage levels. Higher prevalence in simulations with lower treatment coverage led to higher MPSPVLs, replicating effects found in Goodreau et al. (2018b). One likely explanation for this pattern is that there are fewer susceptible individuals in a population with high prevalence, so virulent viruses’ increased transmission probabilities outweigh mortality costs. This effect is weaker than that of shifting transmissions to the acute phase, but as that change was small here, there was little change in MPSPVL.

Model 2A had the same high coital frequency and high proportion of transmissions in the acute phase as Model 1A. The change in transmission function from plateauing to increasing accounted for the smaller amount of variation in proportion of transmissions in the acute phase for this model and the correspondingly smaller upward trend in MPSPVL.

Finally, in Model 2B, low coital frequency and the increasing transmission function combined to lead to little change in acute phase transmissions. Missing that strong effect, changing prevalence between treatment coverage levels led to decreasing MPSPVL with increasing treatment coverage.

### 4.2 Overall virulence differences between models

The trends in virulence change discussed above occur in the context of overall MPSPVL differences between models. These patterns are similar to the effects observed in Goodreau et al.’s (2018b) simulation analysis and are shown in a causal model (Fig. 4b). First, models with shorter relationship durations had higher MPSPVLs, as only virulent viruses were likely to transmit before a partnership dissolved. Second, models with the plateauing transmission function had lower MPSPVLs than those with the increasing transmission function. Presumably, this occurred because increasing SPVL with the plateauing function will increase mortality without increasing probability of transmission, as discussed above.

Model 1A had both factors that lead to lower MPSPVLs being beneficial and evolved in that direction. Model 2B had both factors that lead to higher MPSPVLs being advantageous and evolved correspondingly. The other two models have effects in opposite directions and so remain closer to the initial MPSPVL value.

One possible explanation for the pattern seen in MPSPVL with increasing treatment coverage is simple reversion to the mean: the MPSPVLs in Models 1A and 2B had more extreme values with no treatment and moved closer to the initial mean value with higher treatment coverage. We varied condom use in further simulations to decrease the range of MPSPVL values and concluded that the pattern found is not indicative of this; see Supplementary Appendix for full discussion.

There was suggestive evidence that intermediate treatment intervals may lead to higher MPSPVLs for the highest treatment coverage levels in the fixed interval treatment scheme. One potential interpretation is that there was significant transmission early in infection but treatment began soon enough to limit the trade-off with faster time to AIDS. This effect was not replicated in the explicit testing treatment scheme, possibly because enough agents under this scheme were treated early in their disease course to reduce this effect. However, our uncertainty measures do not exclude the possibility that patterns were similar across both treatment schemes.

Limitations of our analysis include that individuals were perfectly adherent and never ceased treatment. A small number of infections (mean over all simulations = 2.6%) occurred while an agent was virally suppressed. While this is likely to be an overestimation of transmission potential at very low VLs (Rodger et al. 2016), it reflects the values in the transmission functions. Our network model was quite simple, in order to emphasize the effects of relationship duration and coital frequency. We based Models 1A and 2A’s sexual partnership parameters on Herbeck et al.’s (2016) alternate model, which had very high coital frequency, limiting the generalizability of those models to heterosexual populations. Condom use was random and did not impact timing of testing. As this study focuses specifically on the current forms of test-and-treat, it does not provide insight into previously noted discrepancies in SPVL evolution in other very different treatment regimens (Blanquart et al. 2016; Wertheim et al. 2019).

Our results highlight the immense impacts of the exact details of behavior, virological, and intrinsic biological processes in understanding fundamental public health outcomes. By varying sexual behaviors and the transmission function between parameter sets—sexual behaviors that all came from published models and transmission functions estimated from data—we generated huge differences in the effect of increasing treatment coverage on virulence. Transmission function especially had huge effects on prevalence and incidence estimates. Each transmission function has some biological plausibility and, given the ethics of future research, we may never know which more accurately estimates the real-world relationship between VL and probability of transmission. However, this relationship is of fundamental importance in understanding HIV spread and warrants further research. While both treatment and human behavior affect SPVL in line with our previous modeling work (Goodreau et al. 2018b , Stansfield et al. 2019), treatment has the stronger effect. Thoughtfulness and transparency about parameter choices are imperative in modeling studies, as these assumptions can lead to huge outcome differences.

With data-derived behavioral parameters in an MSM population, we found that MPSPVL either does not change or decreases with higher ART coverage. As treatment coverage increases worldwide, it is encouraging that the hypothesized tradeoff between higher treatment prevalence and increasing virulence is not guaranteed. Therefore, public health programs may not need to contend with increasing transmission probability and other consequences of the hypothesized increased virulence. We hypothesize that increasing pre-exposure prophylaxis (PrEP) worldwide will likewise decrease MPSPVL, and in a more straightforward method: more individuals on PrEP will mean fewer susceptible individuals in the population. This will increase the time for an infected person to encounter a susceptible individual and make lower SPVLs with longer disease courses more advantageous for the virus. Although patient outcomes will always come before evolutionary concerns in recommending treatment, our results suggest that vigorous test-and-treat strategies may *not* need to face a tradeoff between increasing treatment and evolution of greater virulence. Instead, a virtuous cycle of increasing treatment and lower HIV virulence may amplify the benefit of test-and-treat programs.

## Supplementary Material

veab011_Supplementary_Data

## Data Availability

The EvoNetHIV R package and simulation scripts are available at github/EvoNetHIV/Test_and_Treat. Simulation data are available on request.
