# Supplementary material for: Test-and-treat coverage and HIV virulence evolution among men who have sex with men
Source: Virus Evol. 2021 Feb 10;7(1):veab011. doi: 10.1093/ve/veab011 (PMC7893213; doi:10.1093/ve/veab011)
Supplement: veab011_Supplementary_Data [file veab011_supplementary_data.docx]

**Test-And-Treat Coverage and HIV Virulence Evolution Among Men Who Have Sex With Men**

Supplementary Appendix

Table of Contents

1. Supplementary Figures 2

1.1 Prevalence, Incidence, & SPVL through Time 2

1.2 Proportion of Transmissions in Acute and AIDS Phases 3

1.3 Explicit Testing Analyses 4

1.4 Fixed Interval Treatment Analyses 6

2. Transmission Function Differences 7

3. Potential Reversion to the Mean 9

4. Model overview 10

5. Sexual network 10

5.1 Empirical data 10

5.2 Network structure 10

6. Sexual behaviors and agent attributes 11

7. HIV transmission 12

7.1 Increasing Function 12

7.2 Plateauing Function 13

8. Set point viral load 14

9. Viral dynamics 15

10. Disease progression 17

11. Vital dynamics 18

11.1 Model initialization 18

11.2 Entries 18

11.3 Exits 19

11.4 Aging 19

# Supplementary Figures

## 1.1 Prevalence, Incidence, & SPVL through Time

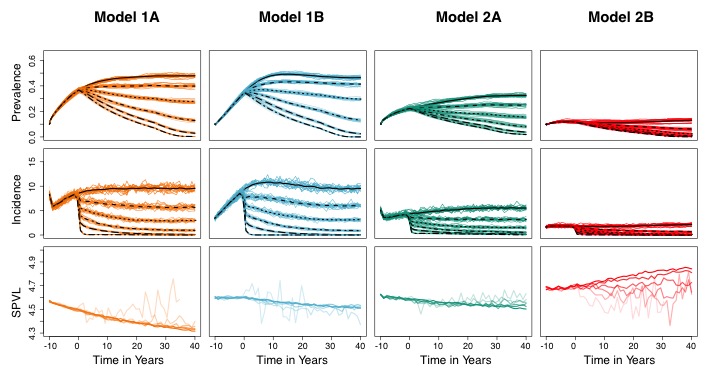


**Figure 1:** Prevalence, Incidence, & MPSPVL by treatment coverage between years -10 to 40. Treatment began year 0. Prevalence & Incidence Plots: Each black line is the mean of 64 simulations. Thin lines show individual simulations. MPSPVL plots: each line is the mean of 64 simulations; lines end when 50% of simulations had no incident infections.

## 1.2 Proportion of Transmissions in Acute and AIDS Phases

**Figure 2:** Mean population proportion of transmissions occurring during the infecting partner’s (A) acute or (B) AIDS phase of those infected in years 20-40. Treatment began in year 0. Each symbol is the mean of 64 simulations. Bars show 95% confidence intervals.

## 1.3 Explicit Testing Analyses

**Figure 3:** Model 1A runs with 1-6 year mean testing intervals. Mean population SPVLs of those infected in years 20-40. Treatment began in year 0. Each symbol is the mean of 16 simulations. Bars show 95% confidence intervals.

**Figure 4:** Model 1B runs with 1-6 year mean testing intervals. Mean population SPVLs of those infected in years 20-40. Treatment began in year 0. Each symbol is the mean of 16 simulations. Bars show 95% confidence intervals.

**Figure 5:** Model 2A runs with 1-6 year mean testing intervals. Mean population SPVLs of those infected in years 20-40. Treatment began in year 0. Each symbol is the mean of 16 simulations. Bars show 95% confidence intervals.

**Figure 6:** Model 2B runs with 1-6 year mean testing intervals. Mean population SPVLs of those infected in years 20-40. Treatment began in year 0. Each symbol is the mean of 16 simulations. Bars show 95% confidence intervals.

## 1.4 Fixed Interval Treatment Analyses

**Figure 7:** Model 1A runs with 1-6 year fixed intervals between infection and treatment initiation. Mean population SPVLs of those infected in years 20-40. Treatment began in year 0. Each symbol is the mean of 16 simulations. Bars show 95% confidence intervals.

**Figure 8:** Model 1B runs with 1-6 year fixed intervals between infection and treatment initiation. Mean population SPVLs of those infected in years 20-40. Treatment began in year 0. Each symbol is the mean of 16 simulations. Bars show 95% confidence intervals.

**Figure 9:** Model 2A runs with 1-6 year fixed intervals between infection and treatment initiation. Mean population SPVLs of those infected in years 20-40. Treatment began in year 0. Each symbol is the mean of 16 simulations. Bars show 95% confidence intervals.

**Figure 10:** Model 2B runs with 1-6 year fixed intervals between infection and treatment initiation. Mean population SPVLs of those infected in years 20-40. Treatment began in year 0. Each symbol is the mean of 16 simulations. Bars show 95% confidence intervals.

# Transmission Function Differences

Some of the difference in shape in transmission functions arises through the paucity of data about transmission at high VLs, since infecting partners in the discordant couples from the source partner studies are unlikely to have extremely high VLs at the time of transmission for a number of reasons. Individuals whose partner had very high SPVLs would likely become infected before being recruited to a discordant partner study. Similarly, individuals would probably be past acute infection at the time of study entry. Sex is also less likely in the late AIDS phase, making transmissions there less probable. This is shown in one partner study as only 3 transmissions and 1% of follow-up time occurred while the infected partner had very high VLs of ≥ 6 log_10_ copies/mL (Lingappa et al., 2010). At the other end of the scale, both the plateauing and increasing transmission functions may overestimate the number of infections at very low VLs; recently (and since both functions were published), the risk of transmission in a discordant couple in which the infected partner is virally suppressed was estimated to have an upper 95% confidence limit of 0.71/100 couple years of follow up for anal sex (Rodger et al., 2016). Small samples sizes (86 and 129 linked transmissions in the cohorts on which the increasing and plateauing functions were based, respectively, (Fideli et al., 2001; Lingappa et al., 2010), methodological differences and noisy data also contribute to the overall differences in function shape.

The plateauing function (Fraser et al., 2007a) is based on data from the Zambian transmission study (Fideli et al., 2001). This study followed 1022 serodiscordant cohabiting couples for between 2 and 67 months between 1994 and 2000; there were 129 linked transmissions. HIV status was assessed quarterly. The data used to create the plateauing transmission function consisted of SPVLs for index cases in partnerships, the overall mean duration of observation, and information about whether the initially uninfected partner became infected (Fraser et al., 2007a). It did not have information about duration of observation of individual partnerships. They created an infection hazard per unit time instead of per serodiscordant sex act, as there were inconsistencies in the reported unprotected sex act frequencies.

The increasing function (Hughes et al., 2012) is based on a prospective study of 3297 serodiscordant couples in the Partners in Prevention Herpes Simplex Virus (HSV)/HIV Transmission Study; there were 86 linked transmissions and data were collected between 2004 and 2007 (Lingappa et al., 2010). HIV positive partners in this study were infected with HSV-2, which does not hinder its generalizability as 60-90% of people living with HIV worldwide are also infected with HSV-2 (Weiss, 2004). This study enrolled couples in eastern and southern Africa and followed them for up to 24 months. HIV tests for negative partners and plasma RNA measurements for positive partners were performed quarterly and number of acts and condom use was assessed monthly. The authors considered many covariates to fit a model that included condom use and time-varying plasma HIV-1 RNA concentration and found a 2.89 increase in the per-act risk of transmission for each log_10_ increase in plasma HIV-1 RNA concentration. They found no evidence of a saturation effect or plateauing relationship between VL and risk of transmission, although only 3 transmissions and 1% of follow-up time occurred while the infected partner had very high VLs of ≥ 6 log_10_ copies/mL.

# 3. Potential Reversion to the Mean

One possible explanation for the pattern seen in MPSPVL with increasing treatment coverage in different models is simple reversion to the mean. Model 1A had the lowest MPSPVL while Model 2B had the highest for the scenario with no treatment (for which details of the care cascade would be irrelevant to explaining differences between models); some form of reversion to the mean with higher coverage could in theory cause the effects seen in these models, and complicate our ability to interpret the trends across the four scenarios. To test this hypothesis, we ran sensitivity analyses varying condom use in order to observe trends in MPSPVL with increasing treatment coverage when the overall MPSPVL levels are more similar (Figure 11). While all main scenarios had 50% condom use, distributed randomly in acts, for this sensitivity analysis we changed Model 2B to have 75% condom use in order to decrease overall MPSPVL and changed the remainder of the models to have 0% condom use to increase it. We found no change in the direction of Model 2B’s trend with lower overall MPSPVL. Model 2A and Model 1B had similar overall MPSPVL levels to Model 2B and showed a more pronounced trend toward higher MPSPVLs with higher treatment coverage than in the main analysis. Only overall MPSPVL in Model 1A did not change substantially with decreased condom use, but neither did its trend with increasing treatment coverage. As the direction of the MPSPVL trends in each model did not change, and Models 1B, 2A, and 2B models now cross each other, we concluded that the pattern of decreasing MPSPVL with increasing coverage in Model 2B is not indicative of simple reversion to the mean. The lack of change in Model 1A may be due to the large number of sex acts and higher chance of transmission of low VL viruses with the plateauing function: if almost all serodiscordant partnerships resulted in transmitting HIV even with low VLs, there would be less scope for change with lower condom use.


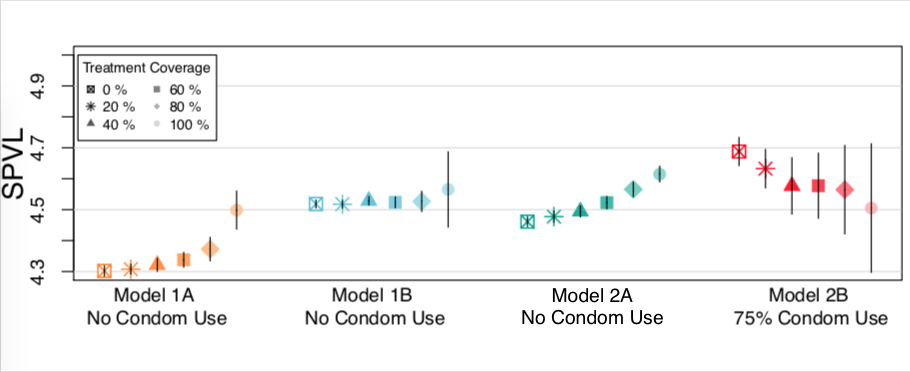


**Figure 11:** Mean population set point viral load trends with changed condom use of those infected in years 20-40. Treatment began in year 0. Each symbol is the mean of 64 simulations. Bars show 95% confidence intervals.

# 4. Model overview

*Note: This and subsequent sections of the appendix are derived from earlier versions of the EvoNetHIV methodological appendix, updated to reflect the parameters and methods specific to this analysis.*

*EvoNetHIV* is written as a series of modules, with multiple options for each module and the option to write additional modules. It also includes over 100 parameters that users can alter, while providing default values for all of those parameters. Here we describe the *EvoNetHIV* components and parameters used in this paper; for more details, see https://github.com/EvoNetHIV/EvoNetHIV. For R scripts to run the models in this paper see https://github/EvoNetHIV/Test_and_Treat. In the description below, all references to VL and SPVL are log_10_ copies/mL, unless otherwise noted. *EvoNetHIV* is programmed in the R software language (R Development Core Team, 2008).

Simulations were conducted on the Hyak supercomputer system at University of Washington, an advanced computational, storage, and networking infrastructure provided by funding through the Student Technology Fee and the Center for Studies in Demography and Ecology.

# 5. Sexual network

## 5.1 Empirical data

Additional details regarding study design and analysis are available in Jenness et al. (2016b) and Goodreau et al. (2017).

## 5.2 Network structure

The sexual network consists of a population of MSM. Parameters include mean momentary degree (0.70, i.e., average number of relationships a man is in at a cross-section of time), coital frequency, and mean relational duration. Two men with incompatible sexual role (i.e., two exclusively insertive men or two exclusively receptive men) are prohibited from forming a partnership. All existing relationships have a constant and equal daily probability of dissolution.

Separable temporal exponential random graph models (STERGMs) (Krivitsky and Handcock, 2014), as implemented in the *statnet* (Handcock et al., 2003) and EpiModel (Jenness et al., 2016a) software suites, were used to estimate the networks. These algorithms also allow us to simulate a dynamic network that maintains our desired network features stochastically, even as the number of men in the network changes, as do their attributes.

**Table 5.1**. Model parameters utilized in network estimation

| **Model parameter** | **Value** | **Source(s) and notes** |
| --- | --- | --- |
| Momentary mean degree | 0.70 | Jenness et al. (2016c) Calculated as the weighted mean of the momentary mean degree of the main, casual, and one-time sexual networks. |
| Sexual role proportions | Exclusively insertive: 24%  Exclusively receptive: 27%  Versatile: 49% | Goodreau et al. (2018); based on data from the InvolveMENt (Sullivan et al., 2015) and MAN Project (Hernandez-Romieu et al., 2015) studies |
| Relationship duration (months), Models 1A & 2A | 30 | Herbeck et al. (2016)’s alternative model |
| Relationship duration (months), Models 1B & 2B | 3.3 | Calculated as a compromise between the weighted mean of the mean relationship durations of the main, casual, and one-time sexual networks (54 days) and the main and casual sexual networks (212 days) from Goodreau et al. (2018). |

The parameter estimates obtained at model initialization are then used in each subsequent time step of the simulation to update the network configuration. We use the offset method of Krivitsky et al. (2011) to account for the changing size of the network as the simulation progresses.

# 6. Sexual behaviors and agent attributes

Coital acts are determined among agents in a serodiscordant relationship at each time step. Among these partnerships, the number of coital acts per partnership at a given time step is assigned according to a Poisson draw with mean of 0.2 or 1 acts/day, which terminated in late-stage AIDS. Circumcision status is assigned to agents at model entry with 85% probability.

**Table 6.1**. Model parameters specifying sexual behaviors and agent attributes

| **Model parameter** | **Value** | **Source(s) and notes** |
| --- | --- | --- |
| Mean sex acts per day,  Models 1A & 2A | 1.0 | Herbeck et al. (2016)’s alternative model |
| Mean sex acts per day, Models 1B & 2B | 0.20 | 0.20 is a reanalysis of parameters in a previous study of MSM with multiple relational types (Goodreau et al., 2017) for a single relational type; based on data from the InvolveMENt (Sullivan et al., 2015) and MAN Project (Hernandez-Romieu et al., 2015) studies |
| Circumcision probability | 0.85 | Mean from two previous modeling studies among MSM. Jenness et al. (2016c); Goodreau et al. (2012) |

# 7. HIV transmission

## 7.1 Increasing Function

The risk of HIV transmission to the uninfected agent is determined for each sex act according to characteristics of the sexual act and characteristics of the agents engaged in the sexual act. We begin with a model that provides a functional form that includes numerous covariates, and relative risk estimates for those covariates (Hughes et al., 2012). However, the published results did not include an estimate for the base value of the function ($\lambda)$, which we obtained directly from the authors. Moreover, that model was specified for penile-vaginal sex, whereas our model considers penile-anal sex. To identify relative risks for these two act types by role, we turned to a meta-analysis (Patel et al., 2014), which provides risk estimates for vaginal receptive (8 per 10,000 exposures), vaginal insertive (4 per 10,000 exposures), anal receptive (138 per 10,000 exposures), and anal insertive intercourse (11 per 10,000 exposures). However, each of these risks was irrespective of circumcision status of the insertive partner. Because our model explicitly accounts for reduced risk among circumcised males, we performed back-calculations accounting for prevalence of circumcision in United States males to estimate the risk for an uncircumcised male of vaginal insertive (8 per 10,000 exposures) and anal insertive intercourse (23 per 10,000 exposures). From these values, we calculated the risk of insertive and receptive anal intercourse relative to vaginal intercourse.

Combing these pieces, the probability of transmission is calculated for each sexual act that occurs in a serodiscordant relationship, as:

$$P\left( transmission \right)=1-{(1-\lambda)}^{e^{X\beta}}$$

where

$$X\beta=\ln\left( 2.89 \right)*\left( viral load-4.0 \right)+\ln\left( 2.9 \right)*insertive+\ln\left( 17.3 \right)*receptive+\ln\left( 0.53 \right)*circumcised+\ln\left( 0.22 \right)*condom$$

**Table 7.1**. Model parameters determining HIV transmission probability per serodiscordant sexual act

| **Model parameter** | **Value** | **Source(s) and notes** |
| --- | --- | --- |
| Per-act infectivity (λ) | 0.000247 | J. Hughes, personal communication, November 14, 2014 |
| Viral load base | 4.0 | J. Hughes, personal communication, November 14, 2014 |
| Relative risk of log_10_ increase in viral load | 2.89 | Hughes et al. (2012) |
| Relative risk of condom use | 0.22 | Hughes et al. (2012) |
| Relative risk of circumcision | 0.53 | Hughes et al. (2012) |
| Relative risk of insertive anal intercourse | 2.9 | Derived (see text) from Patel et al. (2014) |
| Relative risk of receptive anal intercourse | 17.3 | Derived (see text) from Patel et al. (2014) |

## 7.2 Plateauing Function

The Plateauing Function followed the model from Fraser et al. (2007a). This is an increasing Hill function that depends on the maximum infection rate per year (0.317), the slope of increasing infectiousness by viral load (1.02), and the viral load at which infectiousness is half of the maximum (13,938 copies per mL). We back-calculated an approximate per-act maximum transmission probability (0.002) from Fraser’s annual estimate based on previous work (Herbeck et al., 2014). That model was specified for penile-vaginal sex, whereas our model considers penile-anal sex. To identify relative risks for these two act types by role, we turned to Patel et al. (2014), which provides risk estimates from a meta-analysis for vaginal receptive (8 per 10,000 exposures), vaginal insertive (4 per 10,000 exposures), anal receptive (138 per 10,000 exposures), and anal insertive intercourse (11 per 10,000 exposures). However, each of these risks was irrespective of circumcision status of the insertive partner. Because our model explicitly accounts for reduced risk among circumcised males, we performed back-calculations accounting for prevalence of circumcision in United States males to estimate the risk for an uncircumcised male of vaginal insertive (8 per 10,000 exposures) and anal insertive intercourse (23 per 10,000 exposures). From these values, we calculated the risk of insertive and receptive anal intercourse relative to vaginal intercourse.

$$P\left( transmission \right)= \beta(V)=\frac{\left[ 0.002*\left( viral load^{1.02} \right) \right]}{\left( {13938}^{1.02} \right)+\left( viral load^{1.02} \right)} * \left( condom * 0.22 \right)*$$

$$(insertive * 2.9) * (receptive * 17.3) * (circumcised * 0.53)$$

**Table 7.2**. Model parameters determining HIV transmission probability per serodiscordant coital act

| **Model parameter** | **Value** | **Source(s) and notes** |
| --- | --- | --- |
| Maximum infection rate per year | 0.317 | Fraser et al. (2007a) |
| Slope of increasing infectiousness by viral load | 1.02 | Fraser et al. (2007a) |
| Viral load at which infectiousness is half of the maximum | 13,938 copies per mL | Fraser et al. (2007a) |
| Per-act maximum transmission probability (approximate) | 0.002 | Back-calculated from Fraser et al. (2007a) in Herbeck et al. (2014) |
| Relative risk of circumcision | 0.53 | Hughes et al. (2012) |
| Relative risk of insertive anal intercourse | 2.9 | Derived from Patel et al. (2014) (see text) |
| Relative risk of receptive anal intercourse | 17.3 | Derived from Patel et al. (2014) (see text) |

# 8. Set point viral load

Set point viral load (SPVL) in infected agents at model initialization is generated as a combination of viral (viral genotype) and environmental (a combination of undefined host and non-viral) factors. For infected agent *i* present at the start of the model, the viral contribution to SPVL is drawn from a normal distribution:

$${viral}_{spvl,i}\sim N\left( \mu_{spvl,t_{0}},\sigma_{viral,t_{0}}^{2} \right)$$

$\mu_{spvl,t_{0}}=4.5$ log_10_ copies/mL

$\sigma_{viral,t_{0}}^{2}=h^{2} \sigma_{spvl,t_{0}}^{2}$,

where *h^2^* is the heritability coefficient, set here at 0.36, following Hollingsworth et al. (2010), and consistent with the Fraser et al. meta-analysis (2014), and $\sigma_{spvl,t_{0}}^{2}$is the variance of the distribution of SPVL in the initial population at model start. Note that this while the value of *h^2^* is set as a model input and employed to modulate the influence of viral genotype on the similarity in SPVL between transmission pairs, heritability is a population-level measure that can change over time and across populations.

For infections after model start, the viral component for newly infected agent *i* is:

$${viral}_{spvl,i}={viral}_{spvl, infector}+\epsilon$$

where $\epsilon$ is the assumed normally distributed stochastic mutational variance, $\epsilon\sim N\left( 0, 1e-4 \right)$.

The stochastic environmental contribution is normally distributed and is calculated similarly for the initial population and subsequent infections:

$${env}_{spvl,i}\sim N\left( 0,\sigma_{env}^{2} \right)$$

$\sigma_{env}^{2}=\left( 1-h^{2} \right)\sigma_{spvl,t_{0}}^{2}$.

SPVL is then the sum of the viral and environmental contributions, constrained to a minimum value of 2 log_10_ copies/mL and a maximum value of 7 log_10_ copies/mL:

$${spvl}_{i}={env}_{spvl,i}+{viral}_{spvl,i}.$$

**Table 8.1**. Model parameters utilized in the assignment of set point viral load

| **Model parameter** | **Value** | **Source(s) and notes** |
| --- | --- | --- |
| Mean log_10_ SPVL at model initialization | 4.5 | Fraser et al. (2007b); Korenromp et al. (2009); (Herbeck et al., 2008) |
| Heritability of SPVL across transmissions (h^2^) | 0.36 | Hollingsworth et al. (2010) |
| Variance of log_10_ SPVL | 0.8 | Herbeck et al. (2012) |
| Mutational variance | 0.01 | There are no published estimates of mutational variance. We have therefore programmed a low value to be conservative and to maintain approximately 0.36 heritability output measure. |

# 9. Viral dynamics

Upon infection, viral load, *V*, grows exponentially at rate *r*_0_ for the first 21 days according to the formula

$$V(t)=V_{0}e^{r_{0}t}$$

where *V*_0_ is the initial value (set to 0.0001 copies/mL) and *t* indicates the number of days since initial infection. Robb et al. (2016) have shown that viral loads during primary infection correlate with SPVL. Thus, we allowed the peak viral load to depend on the agent’s SPVL as follows

$$V_{peak}=4.639+0.495*{log}_{10}(SPVL)$$

where the values of 4.639 and 0.495 are based on regression data given in Robb et al. (2016). We set *r*_0_ = *ln*(*V*_peak_/*V*_0_)/21 in order to obtain peak viral load on day 21. After reaching peak viral load, viral load decays biphasically. The first phase has a duration of 11 days, in which viral load decays linearly according to the following formula:

$$V\left( t \right)=V_{peak}\left( \frac{V_{32}}{V_{peak}} \right)^{\frac{(t-21)}{11}}$$

where viral load at t=32 is a weighted geometric mean of V_adj_peak_ and SPVL:

$$V_{32}={SPVL}^{0.714}*V_{peak}^{0.286}$$

For the remainder of the duration of acute infection, viral load declines linearly until reaching the agent’s SPVL at day 90 of infection. Viral load decay in this phase is calculated by

$$V\left( t \right)=V_{32}\left( \frac{SPVL}{V_{32}} \right)^{\frac{(t-32)}{58}}$$

In the chronic phase of HIV infection, an agent’s viral load increases at a constant annual rate of 0.14 log_e_ copies/mL, calculated as follows

$$V(t)=SPVL*e^{0.14*\frac{t-90}{365}}$$

This trajectory continues until an agent initiates antiretroviral treatment or enters the AIDS stage, defined by CD4 less than 200 cells/mm^3^. During the AIDS stage, the agent’s viral load increases linearly by 1.004112-fold per day:

$$V(t)=1.004112*V(t-1)$$

Viral load in AIDS increases up to a maximum viral load of 6.38 log_10_ copies/mL.

**Table 9.1**. Model parameters utilized in viral load dynamics

| **Model parameter** | **Value** | **Source(s) and notes** |
| --- | --- | --- |
| Viral load at day 0 of infection | 0.0001 | Model-calibrated to replicate viral dynamics in Lindback et al. (2000) |
| r_0_ | 1.19367006 | Model-calibrated to replicate viral dynamics in Lindback et al. (2000) |
| Duration of exponential viral growth | 21 days | Lindback et al. (2000) |
| Duration of phase 1 decay | 11 days | Lindback et al. (2000) |
| Duration of phase 2 decay | 58 days | Lindback et al. (2000) |
| Duration of acute infection | 90 days | Fiebig et al. (2003) |
| Viral load progression rate, natural log | 0.14 | Geskus et al. (2007) |
| Maximum viral load in AIDS (CD4<200) | 2.4x10^6^ copies/mL = 6.38 log_10_ copies / mL | Piatak et al. (1993) |

# 10. Disease progression

CD4 values determine the additional risk of death among infected agents. Values are categorized as CD4 ≥ 500 cells/mm^3^, 500 < CD4 ≤ 350, 350 < CD4 ≤ 200, and CD4 < 200. Agents are assigned a CD4 category probabilistically according to their set point viral load (Cori et al. (2015); Table 7.1). No agents are assigned a CD4 category of less than 200 cells/mm^3^ upon initial infection.

**Table 10.1**. Probability of assignment to CD4 category stratified by set point viral load

| Set point viral load (log_10_ copies/mL) | CD4 level (cells/mm^3^) | | |
| --- | --- | --- | --- |
|  | ≥ 500 | 350 – 500 | 200 – 350 |
| [2.0, 3.0] | 0.88 | 0.12 | 0.00 |
| (3.0, 3.5] | 0.87 | 0.12 | 0.01 |
| (3.5, 4.0] | 0.85 | 0.12 | 0.03 |
| (4.0, 4.5] | 0.78 | 0.19 | 0.03 |
| (4.5, 5.0] | 0.73 | 0.21 | 0.05 |
| (5.0, 5.5] | 0.71 | 0.25 | 0.04 |
| (5.5, 6.0] | 0.64 | 0.27 | 0.09 |
| (6.0, 6.5] | 0.00 | 0.00 | 1.00 |
| (6.5, 7.0] | 0.00 | 0.00 | 1.00 |

We note that the two highest categories are included for the sake of completion, so that any individual who does evolve into this zone will have an associated CD4 value. However, these persons are very rare in the model and die quickly, limiting the persistence of their viral genotype in the population.

In the absence of antiretroviral treatment, infected agents progress through CD4 categories probabilistically according to an exponential distribution with mean *p*^-1^, where *p* is the inverse of the mean amount of time that an individual remains in a specified CD4 category. The mean duration of time in each CD4 category is determined by SPVL (Cori et al. (2015) and personal communication; Table 7.2).

**Table 10.2**. Mean time (in years) spent in each CD4 category stratified by set point viral load

| Set point viral load (log_10_ copies/mL) | CD4 level (cells/mm^3^) | | | |
| --- | --- | --- | --- | --- |
|  | ≥ 500 | 350 – 500 | 200 – 350 | < 200 |
| [2.0, 3.0] | 6.08 | 5.01 | 3.60 | 4.67 |
| (3.0, 3.5] | 4.69 | 2.52 | 3.68 | 4.11 |
| (3.5, 4.0] | 3.94 | 4.07 | 2.38 | 3.54 |
| (4.0, 4.5] | 2.96 | 3.09 | 3.81 | 2.98 |
| (4.5, 5.0] | 2.25 | 2.32 | 3.21 | 2.42 |
| (5.0, 5.5] | 1.47 | 1.55 | 2.27 | 1.86 |
| (5.5, 6.0] | 0.95 | 1.19 | 1.00 | 1.29 |
| (6.0, 6.5] | 0.32 | 0.59 | 0.68 | 0.73 |
| (6.5, 7.0] | 0.30 | 0.46 | 0.37 | 0.17 |

# 11. Vital dynamics

## 11.1 Model initialization

The epidemic model is initialized with a population size of 10,000 agents. The initial age distribution of model agents is obtained for United States males ages 18-85 from Centers for Disease Control and Prevention (CDC) Wide-ranging Online Data for Epidemiologic Research (WONDER) data for the years 1999-2003 (Centers for Disease Control and Prevention, 2015). This age distribution was used in a model without treatment, reflecting the high AIDS mortality rate observed in the first two decades of the AIDS epidemic, until reaching an equilibrium with respect to age. This equilibrium age distribution is scaled to the age range of 18-55, such that the sum of proportions of agents in each age category is equal to 1. The age of each agent is then randomly assigned with probability of a given age equal to the proportion of the scaled equilibrium U.S. male population of that age.

## 11.2 Entries

The number of entries (births) into the model at each time step is determined by a Poisson draw from a distribution with mean 1.37. This distribution results in approximately 1% annual population growth when all of the default Evonet parameters are used. Each new agent enters the model uninfected with age 18.

## 11.3 Exits

Age-specific annual mortality rates for US males ages 18-55 were obtained from the CDC WONDER database for the years 1999-2003 (Centers for Disease Control and Prevention, 2015). We converted these annual mortality rates to daily probabilities.

Natural deaths occur according to each agent’s age-specific probability of death, and are determined probabilistically by a random draw from a uniform distribution on [0, 1]. HIV-infected agents with CD4 greater than 200 cells/mm^3^ have an increased probability of death that is dependent on their CD4 category.

Deaths due to AIDS occur when an agent’s time in CD4 category 4 (CD4 < 200 cells/mm^3^) is completed according to disease progression described in Section 7.

## 11.4 Aging

Each agent’s age is incremented by 1/365 at each time step.

**Table 11.1**. Model parameters governing vital dynamics

| **Model parameter** | **Value** | **Source(s) and notes** |
| --- | --- | --- |
| Initial population size | 10,000 | NA |
| λ for model entries (births) | 1.37 | Model-calibrated to produce 1% annual growth |
| Minimum age | 18 | NA |
| Maximum age | 55 | NA |
| Age distribution | 0.0450, 0.0440, 0.0430, 0.0420, 0.0410, 0.0400, 0.0390, 0.0380, 0.0370, 0.0360, 0.0350, 0.0340,  0.0330, 0.0320, 0.0310, 0.0300, 0.0290, 0.0280, 0.0270, 0.0260, 0.0250, 0.0240, 0.0230, 0.0220, 0.0210, 0.0200, 0.0190, 0.0180, 0.0170, 0.0160, 0.0150, 0.0140, 0.0130, 0.0120, 0.0110, 0.0100,  0.0090 | Modified from CDC WONDER (Centers for Disease Control and Prevention, 2015) |
| Age-specific annual mortality rates | 0.0011, 0.0012, 0.0013, 0.0014, 0.0014, 0.0014, 0.0014, 0.0014, 0.0014, 0.0014, 0.0014, 0.0014, 0.0014, 0.0015, 0.0015, 0.0016, 0.0016, 0.0017, 0.0018, 0.0019, 0.0021, 0.0022, 0.0024, 0.0026, 0.0028, 0.0030, 0.0033, 0.0036, 0.0039, 0.0043, 0.0046, 0.0050, 0.0055, 0.0059, 0.0064, 0.0069, 0.0074 | CDC WONDER (Centers for Disease Control and Prevention, 2015) |
| Additional probability of death with CD4 > 500 cells/mm^3^ | 0.0000112 per day | The values in CASCADE, 2011 (Writing Committee for the CASCADE Collaboration, 2011) are for men with mean age 30. Rates presented here therefore subtract 0.0014, the natural mortality rate for North American males aged 30 (Centers for Disease Control and Prevention, 2015), to estimate an excess death rate associated with this CD4 category. |
| Additional probability of death with CD4 350-500 cells/mm^3^ | 0.0000148 per day | See note above |
| Additional probability of death with CD4 200-350 cells/mm^3^ | 0.0000333 per day | See note above |

References

Centers for Disease Control and Prevention, 2015. Wide-ranging Online Data for Epidemiologic Research.

Cori, A., Pickles, M., van Sighem, A., Gras, L., Bezemer, D., Reiss, P., Fraser, C., 2015. CD4(+) cell dynamics in untreated HIV-1 infection: overall rates, and effects of age, viral load, sex and calendar time. Aids 29, 2435-2446.

Fideli, U.S., Allen, S.A., Musonda, R., Trask, S., Hahn, B.H., Weiss, H., Mulenga, J., Kasolo, F., Vermund, S.H., Aldrovandi, G.M., 2001. Virologic and immunologic determinants of heterosexual transmission of human immunodeficiency virus type 1 in Africa. AIDS research and human retroviruses 17, 901-910.

Fiebig, E.W., Wright, D.J., Rawal, B.D., Garrett, P.E., Schumacher, R.T., Peddada, L., Heldebrant, C., Smith, R., Conrad, A., Kleinman, S.H., Busch, M.P., 2003. Dynamics of HIV viremia and antibody seroconversion in plasma donors: implications for diagnosis and staging of primary HIV infection. Aids 17, 1871-1879.

Fraser, C., Hollingsworth, T.D., Chapman, R., de Wolf, F., Hanage, W.P., 2007a. Variation in HIV-1 set-point viral load: epidemiological analysis and an evolutionary hypothesis. Proc Natl Acad Sci U S A 104, 17441-17446.

Fraser, C., Hollingsworth, T.D., Chapman, R., de Wolf, F., Hanage, W.P., 2007b. Variation in HIV-1 set-point viral load: Epidemiological analysis and an evolutionary hypothesis. Proceedings of the National Academy of Sciences of the United States of America 104, 17441-17446.

Geskus, R.B., Prins, M., Hubert, J.B., Miedema, F., Berkhout, B., Rouzioux, C., Delfraissy, J.F., Meyer, L., 2007. The HIV RNA setpoint theory revisited. Retrovirology 4.

Goodreau, S.M., Carnegie, N.B., Vittinghoff, E., Lama, J.R., Sanchez, J., Grinsztejn, B., Koblin, B.A., Mayer, K.H., Buchbinder, S.P., 2012. What drives the US and Peruvian HIV epidemics in men who have sex with men (MSM)? Plos One 7, e50522.

Goodreau, S.M., Rosenberg, E.S., Jenness, S.M., Luisi, N., Stansfield, S.E., Millett, G.A., Sullivan, P.S., 2017. Sources of racial disparities in HIV prevalence in men who have sex with men in Atlanta, GA, USA: a modelling study. Lancet HIV 4, e311-e320.

Goodreau, S.M., Rosenberg, E.S., Jenness, S.M., Luisi, N., Stansfield, S.E., Millett, G.A., Sullivan, P.S., 2018. Isolating the sources of racial disparities in HIV prevalence among men who have sex with men (MSM) in Atlanta, GA: A modeling study. The Lancet HIV.

Handcock, M.S., Hunter, D.R., Butts, C.T., Goodreau, S.M., Morris, M., 2003. statnet: Software tools for the Statistical Modeling of Network Data.

Herbeck, J.T., Gottlieb, G.S., Li, X., Hu, Z., Detels, R., Phair, J., Rinaldo, C., Jacobson, L.P., Margolick, J.B., Mullins, J.I., 2008. Lack of evidence for changing virulence of HIV-1 in North America. PLoS One 3, e1525.

Herbeck, J.T., Mittler, J.E., Gottlieb, G.S., Goodreau, S.M., Murphy, J.T., Cori, A., Pickles, M., Fraser, C., 2016. Evolution of HIV virulence in response to widespread scale up of antiretroviral therapy: a modeling study. Virus evolution 2, vew028.

Herbeck, J.T., Mittler, J.E., Gottlieb, G.S., Mullins, J.I., 2014. An HIV epidemic model based on viral load dynamics: value in assessing empirical trends in HIV virulence and community viral load. PLoS Comput Biol 10, e1003673.

Herbeck, J.T., Muller, V., Maust, B.S., Ledergerber, B., Torti, C., Di Giambenedetto, S., Gras, L., Gunthard, H.F., Jacobson, L.P., Mullins, J.I., Gottlieb, G.S., 2012. Is the virulence of HIV changing? A meta-analysis of trends in prognostic markers of HIV disease progression and transmission. AIDS 26, 193-205.

Hernandez-Romieu, A.C., Sullivan, P.S., Rothenberg, R., 2015. Heterogeneity of HIV Prevalence Among the Sexual Networks of Black and White Men Who Have Sex With Men in Atlanta: Illuminating a Mechanism for Increased HIV Risk for Young Black Men Who Have Sex With Men. Sexually Transmitted Diseases 42, 505-512.

Hollingsworth, T.D., Laeyendecker, O., Shirreff, G., Donnelly, C.A., Serwadda, D., Wawer, M.J., Kiwanuka, N., Nalugoda, F., Collinson-Streng, A., Ssempijja, V., Hanage, W.P., Quinn, T.C., Gray, R.H., Fraser, C., 2010. HIV-1 transmitting couples have similar viral load set-points in Rakai, Uganda. Plos Pathog 6, e1000876.

Hughes, J.P., Baeten, J.M., Lingappa, J.R., Magaret, A.S., Wald, A., de Bruyn, G., Kiarie, J., Inambao, M., Kilembe, W., Farquhar, C., Celum, C., Partners Prevention, H.S.V.H.I.V.T., 2012. Determinants of Per-Coital-Act HIV-1 Infectivity Among African HIV-1-Serodiscordant Couples. Journal of Infectious Diseases 205, 358-365.

Jenness, S.M., Goodreau, S.M., Morris, M., 2016a. EpiModel: Mathematical Modeling of Infectious Disease.

Jenness, S.M., Goodreau, S.M., Rosenberg, E., Beylerian, E.N., Hoover, K.W., Smith, D.K., Sullivan, P., 2016b. Impact of the Centers for Disease Control's HIV Preexposure Prophylaxis Guidelines for Men Who Have Sex With Men in the United States. J Infect Dis 214, 1800-1807.

Jenness, S.M., Goodreau, S.M., Rosenberg, E., Beylerian, E.N., Hoover, K.W., Smith, D.K., Sullivan, P., 2016c. Impact of the Centers for Disease Control's HIV Preexposure Prophylaxis Guidelines for Men Who Have Sex With Men in the United States. J Infect Dis.

Korenromp, E.L., Williams, B.G., Schmid, G.P., Dye, C., 2009. Clinical Prognostic Value of RNA Viral Load and CD4 Cell Counts during Untreated HIV-1 Infection-A Quantitative Review. Plos One 4.

Krivitsky, P.N., Handcock, M.S., 2014. A separable model for dynamic networks. Journal of the Royal Statistical Society Series B-Statistical Methodology 76.

Krivitsky, P.N., Handcock, M.S., Morris, M., 2011. Adjusting for network size and composition effects in exponential-family random graph models. Statistical Methodology 8, 319-339.

Lindback, S., Karlsson, A.C., Mittler, J., Blaxhult, A., Carlsson, M., Briheim, G., Sonnerborg, A., Gaines, H., Karolinska Institutet Primary, H.I.V., 2000. Viral dynamics in primary HIV-1 infection. Aids 14, 2283-2291.

Lingappa, J.R., Baeten, J.M., Wald, A., Hughes, J.P., Thomas, K.K., Mujugira, A., Mugo, N., Bukusi, E.A., Cohen, C.R., Katabira, E., Ronald, A., Kiarie, J., Farquhar, C., Stewart, G.J., Makhema, J., Essex, M., Were, E., Fife, K.H., de Bruyn, G., Gray, G.E., McIntyre, J.A., Manongi, R., Kapiga, S., Coetzee, D., Allen, S., Inambao, M., Kayitenkore, K., Karita, E., Kanweka, W., Delany, S., Rees, H., Vwalika, B., Magaret, A.S., Wang, R.S., Kidoguchi, L., Barnes, L., Ridzon, R., Corey, L., Celum, C., 2010. Daily acyclovir for HIV-1 disease progression in people dually infected with HIV-1 and herpes simplex virus type 2: a randomised placebo-controlled trial. Lancet (London, England) 375, 824-833.

Patel, P., Borkowf, C.B., Brooks, J.T., Lasry, A., Lansky, A., Mermin, J., 2014. Estimating per-act HIV transmission risk: a systematic review. AIDS 28, 1509-1519.

Piatak, M., Saag, M.S., Yang, L.C., Clark, S.J., Kappes, J.C., Luk, K.C., Hahn, B.H., Shaw, G.M., Lifson, J.D., 1993. High levels of HIV-1 in plasma during all stages of infection determined by competitive PCR. Science 259, 1749-1754.

R Development Core Team, 2008. R: A language and environment for statistical computing.

Robb, M.L., Eller, L.A., Kibuuka, H., Rono, K., Maganga, L., Nitayaphan, S., Kroon, E., Sawe, F.K., Sinei, S., Sriplienchan, S., Jagodzinski, L.L., Malia, J., Manak, M., de Souza, M.S., Tovanabutra, S., Sanders-Buell, E., Rolland, M., Dorsey-Spitz, J., Eller, M.A., Milazzo, M., Li, Q., Lewandowski, A., Wu, H., Swann, E., O'Connell, R.J., Peel, S., Dawson, P., Kim, J.H., Michael, N.L., Team, R.V.S., 2016. Prospective Study of Acute HIV-1 Infection in Adults in East Africa and Thailand. New England Journal of Medicine 374, 2120-2130.

Rodger, A.J., Cambiano, V., Bruun, T., Vernazza, P., Collins, S., van Lunzen, J., Corbelli, G.M., Estrada, V., Geretti, A.M., Beloukas, A., Asboe, D., Viciana, P., Gutierrez, F., Clotet, B., Pradier, C., Gerstoft, J., Weber, R., Westling, K., Wandeler, G., Prins, J.M., Rieger, A., Stoeckle, M., Kummerle, T., Bini, T., Ammassari, A., Gilson, R., Krznaric, I., Ristola, M., Zangerle, R., Handberg, P., Antela, A., Allan, S., Phillips, A.N., Lundgren, J., 2016. Sexual Activity Without Condoms and Risk of HIV Transmission in Serodifferent Couples When the HIV-Positive Partner Is Using Suppressive Antiretroviral Therapy. Jama 316, 171-181.

Sullivan, P.S., Rosenberg, E.S., Sanchez, T.H., 2015. Explaining racial disparities in HIV incidence in black and white men who have sex with men in Atlanta, GA: a prospective observational cohort study. Annals of Epidemiology 25, 445-454.

Weiss, H., 2004. Epidemiology of herpes simplex virus type 2 infection in the developing world. Herpes : the journal of the IHMF 11 Suppl 1, 24a-35a.

Writing Committee for the CASCADE Collaboration, 2011. Timing of HAART Initiation and Clinical Outcomes in Human Immunodeficiency Virus Type 1 Seroconverters. Archives of Internal Medicine 171, 1560-1569.
